# Retraction Note: Stay-at-home policy is a case of exception fallacy: an internet-based ecological study

**DOI:** 10.1038/s41598-021-03250-7

**Published:** 2021-12-14

**Authors:** R. S. Savaris, G. Pumi, J. Dalzochio, R. Kunst

**Affiliations:** 1 School of Medicine, Department of Obstetrics and Gynecology, Universidade Federal Do Rio Grande Do Sul, Rua Ramiro Barcelos 2400, Porto Alegre, RS CEP 90035‑003 Brazil; 2 Mathematics and Statistics Institute and Programa de Pós‑Graduação em Estatística, Universidade Federal Do Rio Grande Do Sul, 9500, Bento Gonçalves Avenue, Porto Alegre, RS 91509‑900 Brazil; 3 Applied Computing Graduate Program, University of Vale Do Rio Dos Sinos (UNISINOS), Av. Unisinos, 950, São Leopoldo, RS 93022‑750 Brazil; 4 Serv. Ginecologia E Obstetrícia, Hospital de Clínicas de Porto Alegre, Rua Ramiro Barcelos 2350, Porto Alegre, RS CEP 90035‑903 Brazil; 5 Postgraduate of BigData, Data Science and Machine Learning Course, Unisinos, Porto Alegre, RS Brazil

Retraction of: *Scientific Reports* 10.1038/s41598-021-84092-1, published online 05 March 2021

The Editors have retracted this Article.

Following publication of this Article concerns have been raised about the methodological approach developed by the Authors to evaluate the impact of stay-at-home policies on the reduction of COVID-19-related fatalities. In particular, Meyerowitz-Katz et al.^1^ show that the approach fails to detect any signal when tested on a synthetic dataset where the ground truth is known, and under specific cases of data subsetting. In addition, Meyerowitz-Katz et al.^1^ failed to replicate the original results using a synthetic dataset. These suggest that the false negative rate of the approach is prohibitively high to allow for meaningful conclusions to be drawn regarding the impact of stay-at-home policies on COVID-19 fatality rates. The results of Meyerowitz-Katz et al.^1^ are further confirmed by Góes^2^ who, using a pure correlation analysis, shows that the coefficients for the impact of stay-at-home policies using the methodological approach developed by the Authors can be zero even with diametrically opposing indices of staying-at-home. Given these concerns, the Editors no longer have confidence that the conclusions presented are adequately supported.

R.S. Savaris, G. Pumi, J. Dalzochio and R. Kunst do not agree with this retraction.
